# The Taste of Typeface

**DOI:** 10.1177/2041669515593040

**Published:** 2015-08-31

**Authors:** Carlos Velasco, Andy T. Woods, Sarah Hyndman, Charles Spence

**Affiliations:** Department of Experimental Psychology, University Oxford, UK; Imagineering Institute, Nusajaya, Johor, Malaysia; Department of Experimental Psychology, University Oxford, UK; Xperiment, Surrey, UK; Type Tasting, London, UK; Department of Experimental Psychology, University Oxford, UK

**Keywords:** typeface, taste, crossmodal correspondences, affective correspondences, design

## Abstract

Previous research has demonstrated that typefaces can convey meaning over-and-above the actual semantic content of whatever happens to be written. Here, we demonstrate for the first time that people match basic taste words (sweet, sour, salty, and bitter) to typefaces varying in their roundness versus angularity. In Experiment 1, the participants matched rounder typefaces with the word “sweet,” while matching more angular typefaces with the taste words “bitter,” “salty,” and “sour.” Experiment 2 demonstrates that rounder typefaces are liked more and are judged easier to read than their more angular counterparts. We conclude that there is a strong relationship between roundness/angularity, ease of processing, and typeface liking, which in turn influences the correspondence between typeface and taste. These results are discussed in terms of the notion of affective crossmodal correspondences.

## Introduction

Recently, a growing body of research has demonstrated that people match taste and flavor stimuli (as well as taste or flavor words) to shapes in a manner that is consistent across individuals (e.g., see Wan et al., 2014; see also Spence & Deroy, 2013, for a review). The roundness/angularity (RA) of shapes appears to be a key element in the crossmodal matching of tastes to shapes (Velasco, Woods, Deroy, & Spence, 2015a). Research on the “crossmodal correspondences” (the name given to the initially surprising tendency to match seemingly unrelated information across the senses, see Marks & Mulvenna, 2013; Spence, 2011) between tastes and shapes is of particular interest to designers and marketers, as design elements such as logos, labels, visual imagery, and typefaces can potentially influence a consumer’s expectations concerning food and drink products (see Spence, 2012, for a review).

Typefaces are particularly intriguing in this regard, as they can take on a number of different forms and can convey meaning over-and-above the actual semantic message of the text itself (e.g., Doyle & Bottomley, 2009; Morrison, 1986; Poffenberger & Barrows, 1924; Tannenbaum, Jacobson, & Norris, 1964). Indeed, it has been suggested that the visual features of written words can be processed before their actual meaning and that this can influence subsequent information processing (e.g., Childers & Jass, 2002, see also Celhay, Boyselle, & Cohen, 2015). In the context of consumer behavior, this may be of particular importance given that consumers perceive fonts as being more appropriate for a given product when both the product and the font have the same connotative meaning (e.g., Doyle & Bottomley, 2009, as measured using the semantic differential technique; see Osgood, Suci, & Tannenbaum, 1957). In addition, the semantic appropriateness or congruency of a product has also been shown to influence people’s choice behavior (Doyle & Bottomley, 2004, see also Doyle & Bottomley, 2006; 2011). This, in turn, points to the importance of considering font-product semantic consistency in design. Here, we propose that congruency can also be applied to the relationship between typeface and taste. Design practitioners have already suggested that typefaces may be used to convey information about taste or flavor (Hyndman, 2015).

Research on the topic of crossmodal correspondences suggests that the RA of typefaces can be associated with specific taste words (in a forced choice setting). For example, Velasco, Salgado-Montejo, Marmolejo-Ramos, and Spence (2014) recently reported that fictional packages with a brand name that had a rounder typeface (e.g., Swis721 B1kRnd BT—Black, 44 pt) were more frequently categorized as sweet as compared with packages with a more angular typeface (e.g., Hollywood Hills—Regular, 53 pt), which was more frequently categorized as sour instead. Potentially, the RA dimension studied in research on shape-taste correspondences can be extended to typefaces.

In the present study, we wanted to investigate whether the different design elements of typefaces (RA, bold, italics, and orientation) would influence participants’ associations to specific basic taste words (bitter, salty, sour, and sweet). Based on the aforementioned literature, the clear prediction has, at least, to be that rounder typefaces would be associated with the word sweet. In Experiment 1, we assessed any relationship between taste words (bitter, salty, sour, and sweet) and different typefaces. Experiment 2 was motivated by previous research suggesting that taste or shape may be thought of as affective correspondences (Velasco et al., 2015a), and that there is a relationship between processing fluency and liking (Winkielman & Cacioppo, 2001). Here, we assessed whether the different typefaces would be consistently rated along RA, as well as liking, and ease of processing scales.

## Experiment 1

### Methods and Materials

One hundred and one participants (42 females, mean age = 35.4 years, *SD* age = 11.15, range age = 20–69 years) from the United States took part in this study online through the Adobe Flash-based Xperiment software (http://www.xperiment.mobi, also used in Experiment 2). The participants were recruited using Amazon’s Mechanical Turk in exchange for a payment of 1.00 USD in both Experiments 1 and 2. All were based in the United States, and all agreed to take part in the study after reading a standard consent form. The experiment was reviewed and approved by the Central University Research Ethics Committee at the University of Oxford.

**Figure 1.**
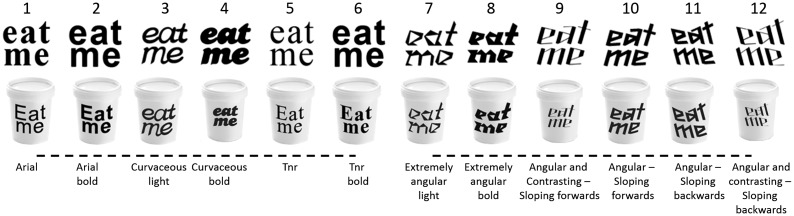
Typeface stimuli presented to the participants in Experiment 1. The typefaces (without the cups) were also used in Experiment 2.

The images of 12 variants of the same text “eat me” were written in different typefaces and were created specifically for the experiments reported here (see Figure 1). Each image was presented in black and white either alone (71 × 71 pixels) or on a receptacle (85 × 116 pixels), giving rise to a total of 24 stimuli. The different typefaces were designed to be distinctively round or angular (Velasco et al., 2014). The typefaces used in the present study are shown in Figure 1. The first two are Arial (Typeface 1) and Arial bold (Typeface 2), while the remaining 10 correspond to designs made by Sarah Hyndman (http://www.sarahhyndman.com/), a professional graphic designer, specifically for the purposes of the present experiment.. Note that the first six typefaces are rounder, while the remaining six are more angular.

**Figure 2.**
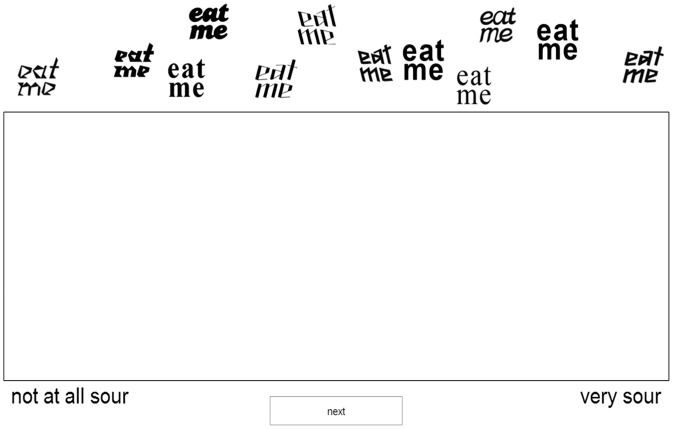
Screen display in one of the experiment trials used in Experiment 1.

Through two tasks, the *task with typeface* and the *task with typeface and cup*, the participants had to arrange the different images of the typeface variants of the text “eat me” alone or on a cup, respectively, in terms of the extent to which they thought they matched bitter, salty, sour, and sweet tastes (see Figure 2, for an example of the task). The tasks were randomized across participants.

Due to a number of repeated measures analysis of variance assumptions being violated (e.g., the data were not normally distributed), the rank-based analysis of variance-type statistic (ATS) was used to analyze the results (see Erceg-Hurn & Mirosevich, 2008), as implemented in the {nparLD} package in R statistical software (see Noguchi, Gel, Brunner, & Konietschke, 2012). The significant main effects were further analyzed with the Wilcoxon signed-rank test with Bonferroni correction. Moreover, because our main interest lay in assessing whether the ratings of each stimulus differed as a function of the taste, we then analyzed the significant interactions of taste and shape with Friedman’s test. After this, multiple comparisons were performed following Siegel and Castellan’s (1988) Formula 7.5a (pp. 180–181), as implemented in {pgirmess} package in R statistical software. Friedman test was also used in Experiment 2.

### Results and Discussion

#### Task with typeface

A 4 (taste words) × 12 (typefaces) ATS was performed. Significant main effects were obtained for taste, *F*_ATS_ (2.54, ∞) = 7.86, *p* < .001, and typeface, *F*_ATS_ (4.36, ∞) = 50.03, *p* < .001. A significant interaction term was also documented, *F*_ATS_ (8.15, ∞) = 54.00, *p* < .001. Bonferroni-corrected Wilcoxon signed-rank tests revealed that sweet ratings were lower than ratings for the other tastes (*p* < .005, for all comparisons). The participants also gave higher ratings to the angular (7–12) as compared with the round (1–6) typefaces (*p* < .001 for all comparisons). Furthermore, Typeface 7, extremely angular light, received higher ratings than Typeface 12, angular and contrasting—sloping backwards (*p* = .02).

**Figure 3.**
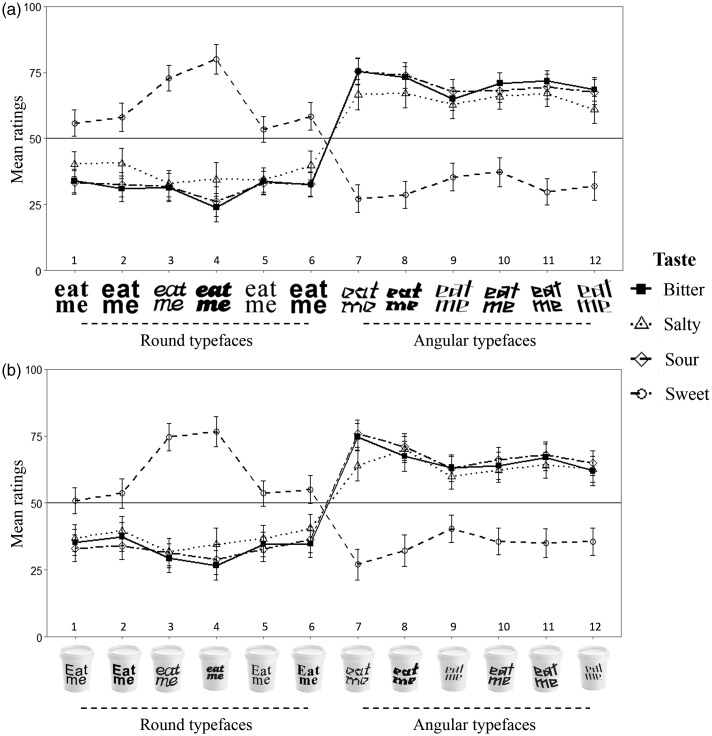
Mean ratings in the task with typeface (a), and the task with typeface and cup (b) in Experiment 1.

As for the interaction term, a significant difference between the different typefaces was found for the bitter, χ^2^(11, *n* = 101) = 455.85, *p* < .001, salty, χ^2^(11, *n* = 101) = 216.14, *p* < .001, sour, χ^2^(11, *n* = 101) = 428.48, *p* < .001, and sweet taste words, χ^2^(11, *n* = 101) = 340.68, *p* < .001. Pairwise comparisons revealed that the participants rated Typefaces 7 to 12 as more bitter, salty, and sour than Typefaces 1 to 6. In addition, the participants rated Typefaces 1, 2, and 6 as significantly sweeter than Typefaces 7 to 12, Typeface 3 as significantly sweeter than 1, 5, and 7 to 12, Typeface 4 as sweeter than 1, 2, and 5 to 12, and Typeface 5 as sweeter than 7 to 9, 11, and 12 (*p* < .05, for all comparisons, see Figure 3(a), for a summary of the results). In other words, the participants rated the angular typefaces as similarly bitter, salty, and sour, as compared with the round typefaces, while the later were associated to sweet, as compared with the angular typefaces.

#### Task with typeface and cup

The analysis revealed significant main effects of taste word, *F*_ATS_ (2.81, ∞) = 5.17, *p* < .005, typeface, *F*_ATS_ (4.10, ∞) = 41.33, *p* < .001, and the interaction between taste word and typeface, *F*_ATS_ (8.73, ∞) = 40.31, *p* < .001. Here, again, the participants' sweet ratings were lower than the ratings of the other tastes (*p* < .01, for all comparisons). Pairwise comparisons performed on the main effect of typeface also revealed that the participants gave higher ratings to the angular (7–12) as compared with the round (1–6) typefaces (*p* < .001, for all comparisons). Moreover, Typeface 7, extremely angular light, received higher ratings than Typeface 9, angular and contrasting—sloping forwards, and Typeface 12, angular and contrasting—sloping backwards (*p* < .01, for both comparisons). As for the interaction term, a significant difference between the different typefaces was observed for the bitter, χ^2^(11, *n* = 101) = 314.64, *p* < .001, salty, χ^2^(11, *n* = 101) = 249.80, *p* < .001, sour, χ^2^(11, *n* = 101) = 374.14, *p* < .001, and sweet taste words, χ^2^(11, *n* = 101) = 254.32, *p* < .001. Pairwise comparisons of this task closely replicated those of the task with just typeface (see Figure 3(b), for a summary of the results). In addition to the results reported for the bitter and sour ratings, Typeface 7 was rated as more bitter than Typeface 12, and as more sour than Typeface 9. As for the sweet ratings, participants rated Typeface 1 as sweeter than Typefaces 7 and 8, and Typefaces 2 and 5 as sweeter than Typefaces 7, 8, and 10 to 12. Typefaces 3 and 4 were rated as sweeter than Typefaces 1, 2, and 5 to 12, and Typeface 6 as sweeter than Typefaces 7, 8, 11, and 12.

The results of Experiment 1 are consistent with previous research in suggesting that round shapes and typefaces are strongly associated with sweet, while angular shapes and typefaces are more strongly associated with the other basic taste words (e.g., Velasco et al., 2014, 2015a, Velasco, Woods, Liu, & Spence, 2015b). One may wonder, however, whether the typefaces used in our first experiment would consistently be rated as more angular or round. In addition, one might also consider whether typeface or taste associations can be thought of as a kind of affective correspondence (Marks, 1978; Velasco et al., 2015a). Typefaces are particularly intriguing in this regard as how easy or difficult they are to process may, in turn, be related to our preference for them (Gump, 2001; Song & Schwarz, 2008); Stimuli that are highly fluent have been shown to be associated with positive affect and more positive judgments (Winkielman, Schwarz, Fazendeiro, & Reber, 2003). Experiment 2 was conducted in order to assess whether the participants would consistently categorize the typefaces on a RA scale. The participants also rated how easy or difficult they found it to read the different typefaces, and how much they liked them.

## Experiment 2

### Methods and Materials

Ninety nine participants (40 females, mean age = 33.8 years, *SD* age = 9.8, range age = 18–62 years) took part in this online study. Following the same procedure used in Experiment 1, the participants were asked to arrange the different typefaces in three different scales or trials: RA, liking, and legibility (easiness or difficulty to read). The three trials were randomly presented.

### Results and Discussion

**Figure 4.**
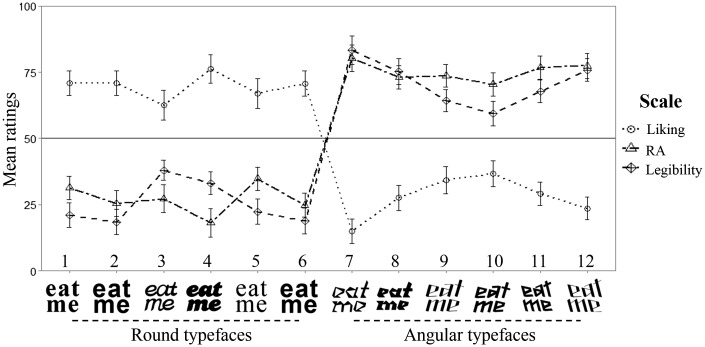
Mean ratings in the task presented in Experiment 2. Note that Typefaces 1 to 6 were generally liked and considered round and easy to read, whereas Typefaces 7 to 12 were generally liked less and considered more angular and less easy to read. RA = roundness/angularity.

The results are summarized in Figure 4. Each variable was analyzed by means of a Friedman’s test. The results revealed a significant difference in liking, χ^2^(11, *n* = 99) = 456.51, *p* < .001, RA, χ^2^(11, *n* = 99) = 539.18, *p* < .001, and legibility ratings, χ^2^(11, *n* = 99) = 560.63, *p* < .001, across typefaces.

Pairwise comparisons revealed that participants liked Typefaces 1 to 6 significantly more than Typefaces 7 to 12 and Typefaces 9 to 11 more than Typeface 7 (*p* < .05, for all comparisons). Moreover, the participants rated Typefaces 7 to 12 as significantly more angular than Typefaces 1 to 6 and Typeface 5 as more angular than Typeface 4 (*p* < .05, for all comparisons). Finally, the participants considered Typefaces 7 to 12 to be less easy to read than Typefaces 1 to 6, Typeface 7 less than Typefaces 9 to 11, Typefaces 8 and 12 less than 10, and Typeface 3 less than Typefaces 1, 2, 5, and 6 (*p* < .05, for all comparisons).

Kendall's Tau correlations between RA, legibility, and liking, as a function of the typeface averages, were also calculated. A significant positive correlation was observed between RA and legibility (*r* = .73, *p* < .001). The more angular the typeface was rated, the less easy it was to read. In addition, significant negative correlations were observed between RA and liking (*r* = −.79, *p* < .001), and between legibility and liking (*r* = −.82, *p* < .001). The more angular the typeface ratings, the less the participants liked it, and the less easy the typeface was to read, the less liked it was.

### Taste or Typeface Correspondence

Based on the results of Experiment 2, further analysis of the data from Experiment 1 was conducted in order to determine whether those typefaces that were considered as round as a whole versus those that were considered as angular would influence taste ratings differently. Here, the data from the two tasks in Experiment 1 (as the results were virtually the same) were combined, and the different taste word scores were averaged as a function of the round (1–6) and angular (7–12) typefaces.^1^ A 2 (RA: round, angular) × 4 (taste: bitter, salty, sour, sweet) ATS revealed a significant effect of RA, *F*_ATS_ (1.00, ∞) = 132.92, *p* < .001, taste, *F*_ATS_ (2.51, ∞) = 11.12, *p* < .001, and the interaction between taste and RA, *F*_ATS_ (2.11, ∞) = 116.44, *p* < .001. Wilcoxon signed-rank tests with Bonferroni correction revealed that the angular typefaces received higher taste ratings than the round typefaces (*p* < .001). Pairwise comparisons also revealed that, overall, sweet ratings were lower compared with the other taste ratings (*p* < .001, for all comparisons). As for the interaction term, a significant difference was found across taste ratings as a function of round, χ^2^(3, *n* = 101) = 111.00, *p* < .001, and angular, χ^2^(3, *n* = 101) = 90.97, *p* < .001, typefaces. The sweet ratings were significantly higher for the round typefaces, and significantly lower for the angular typefaces, as compared with the other tastes (*p* < .05, for all comparisons).

The latter results confirmed what was initially suggested in Experiment 1. The participants associated round typefaces with sweet tastes and angular typefaces with the other tastes. Note that rounder typefaces were liked more and were also considered as being easier to read. In addition, it is well known that people prefer sweet tastes (e.g., Drewnowski, 1997). It would therefore seem like there is an intrinsic relationship between RA, fluency, and liking (cf. Bar & Neta, 2006; Collier, 1996), which, in turn, influences the crossmodal correspondence between such feature and tastes. As Marks (1978, p. 75) puts it some years ago: “It just might be that the affective or hedonic dimension of sensation forms, in part, a basis for qualitative similarities among the senses.”

## General Discussion

The present study was designed to assess the way in which people match shapes to taste words, and typefaces, varying in their RA, to taste words. The results reported here add weight to the notion that “sweet” tends as be associated with round shapes and forms (Velasco et al., 2015a; 2015b). Furthermore, the results also suggest a clear distinction between round and angular typefaces, with the former being liked more, considered as easier to read, and associated with sweet, compared with the latter which were less liked, considered less easy to read, and associated with the other tastes (e.g., sour, salty, and bitter). Overall, these results are consistent with previous research showing that there is no apparent distinction between bitter, salty, and sour, when participants match them to curvy and angular shapes (Velasco et al., 2015b).

Why would people match tastes and typefaces varying in their roundness and angularity? In a recent study, Velasco et al. (2015a) demonstrated that taste hedonics can influence the way in which people match tastes to shapes. In other words, the more that an individual likes a taste, the more they will choose a round shape to match it to, and the less they like it, the more they will tend to associate the taste with an angular shape instead (see also Velasco et al., 2015b). It has been suggested that this may be related to the idea that people prefer round objects, as compared with angular objects (e.g., Bar & Neta, 2006; Westerman et al., 2012).

Crucially, previous research has suggested that the features of typeface convey emotional meaning, with curved typefaces being associated with more positively valenced emotions and angular typefaces being associated with more negatively valenced emotions (Kastl & Child, 1968; Morrison, 1986). This, together with the findings of Velasco et al. (2015a; 2015b), means that it is possible to hypothesize that those who associate curved typefaces with more positively valenced emotions will be more likely to associate them with sweetness, a taste that is known for its positive hedonic effect on humans (Drewnowski, 1997; Steiner, Glaser, Hawilo, & Berridge, 2001).

Therefore, it is possible to think that taste or typeface correspondences may be thought of as a form of affective correspondence (e.g., Collier, 1996; see also Marks, 1996). The idea here is that certain cross-sensory matches may be based on a common feeling elicited by the information presented to two different senses (e.g., music and colors, see Palmer, Schloss, Xu, & Prado-Leon, 2013; or odors and colors, Schifferstein & Tanudjaja, 2004). An alternative explanation that is not necessarily incompatible (it should rather be considered as complementary) with the latter is related to how easy the different typefaces are to process (this is known as “processing fluency”, e.g., Song & Schwartz, 2008). Previous research has shown that processing fluency can result in positive affect (Winkielman & Cacioppo, 2001). That is, stimuli that are easy to process will be experienced as more pleasing and evaluated more positively than those stimuli that are hard to process (see also Winkielman et al., 2003). As sweet is considered a pleasant taste, participants may match it to those typefaces that are easy-to-read and pleasing. The results of the present study therefore reveal that there is a close relationship between typeface RA, easiness to read, and liking, which in turn, may explain the correspondence between taste and typeface RA.

The type of scales that were used in Experiments 1 and 2 may, in part, explain the clear distinction between round and angular typefaces in all variables. It would therefore be interesting to use different scales in future research. It would also be important to assess a participant’s familiarity with the typefaces, as there is a relationship between fluency and familiarity (Westerman, Lanska, & Olds, 2014). While most of the typefaces used here were novel, one may wonder whether they evoke feelings of familiarity and whether familiarity also plays a role on the associations between typeface and taste.

While we did not use actual tastants here, simply by using taste words, it is possible to have an estimate of the way in which people would match the tastes (Velasco et al., 2015a). It would be interesting in future research to test such a hypothesis with, for example, flavors or perfumes. Another interesting direction for future research, and one that would allow for the testing of the hypothesis forwarded here, would be to assess taste or typeface associations across languages. Finally, one may also wonder whether specific semantic contents of words (e.g., cake) written in particular typefaces (round vs. angular) may interact in their association to taste (perhaps in a Stroop-like fashion).

## Note

1 One-sample *t* tests were performed in order to assess whether the different typefaces would differ from the midpoint (50) of the RA, liking, and ease-of-reading scales. As expected, Typefaces 1 to 6 were significantly different from the midpoint toward the round end of the scale and Typefaces 7 to 12 toward the angular end of the scale. Moreover, the round (1–6) typefaces were significantly liked (significantly toward “like”) and considered easy to read (significantly toward “easy”), while the angular typefaces (7–12) were significantly disliked (toward “dislike”) and considered difficult to read (significantly toward difficult, *p* < .01, for all comparisons).
